# Salvia miltiorrhiza bunge increases estrogen level without side effects on reproductive tissues in immature/ovariectomized mice

**DOI:** 10.18632/aging.101145

**Published:** 2016-12-20

**Authors:** Ying Xu, Ting Chen, Xin Li, Ya-kun Qu, Jin-na An, Hong-xia Zheng, Zi-jia Zhang, Na Lin

**Affiliations:** ^1^ Institute of Chinese Materia Medica, China Academy of Chinese Medical Sciences, Beijing 100700, China; ^2^ Institute of Chinese Materia Medica, Shanghai University of Traditional Chinese Medicine, Shanghai 100101, China

**Keywords:** Salvia miltiorrhiza bunge, reproductive target tissue, estrogenic effect, estrogen receptors, estrogen receptor antagonist ICI182, 780

## Abstract

Salvia miltiorrhiza bunge (SM) is a popular herb for alleviating menopausal symptoms, although the scientific evidence of applying SM to estrogen replacement therapy is limited. In this study, we characterized the estrogenic activity of SM using *in vivo* models of immature and ovariectomized (OVX) mice and performed *in vitro* studies focusing on the estrogen receptor (ER) pathway for further molecular characterizations. SM treatments demonstrated significant estrogenic activity by promoting the development of uterus and vagina in immature mice, restoring the estrus cycle and reversing the atrophy of reproductive tissues in OVX mice, as well as increasing the expressions of ERα and ERβ at protein and mRNA level in the reproductive tissues. Meanwhile, SM significantly increased estradiol in serum, and decreased follicle-stimulating hormone (FSH) and luteinizing hormone (LH) in the circulation of immature and OVX mice. SM could stimulate the binding effect of ERα and ERβ, and significantly induce ERα/β-estrogen response element (ERE) luciferase reporter gene expression. All these activities were inhibited by the ER antagonist ICI182, 780. This study demonstrates SM exerts estrogenic effects by stimulating biosynthesis of estrogen and increasing ERs in target tissues without side effects on reproductive tissues and through ER-ERE-dependent pathway.

## INTRODUCTION

In postmenopausal women, ovarian estrogen deficiency results in a series of short-time postmenopausal symptoms such as hot flashes, sweating, anxiety, and mood swings as well as an increased risk for many chronic health problems such as cardiovascular diseases and osteoporosis. Hormonal replacement therapy (HRT) has been long considered to help protect women against these aging-associated symptoms or diseases [1, 2]. However, numerous investigations have indicated that HRT significantly increased the risk of gynecological tumor and other undesirable side effects, including breast tenderness and uterine bleeding [3, 4]. Therefore, many researchers have explored to use phytoestrogens from herbal medicines as alternatives [5]. Phyto-estrogens are similar to mammalian estrogens both structurally and functionally, and they have advantages of lower side effects compared with synthetic HRT [6, 7]. Traditional Chinese Medicines (TCM), as a new phytoestrogens resource, has already attracted the attention of researchers.

Salvia miltiorrhiza Bunge (SM), known as Danshen in China, is one of the most popular TCM. It has been used for more than a millennium in Asian countries, especially in China, Japan, and Korea [8]. SM has been widely used in clinical practice for the prevention of cardiac diseases, arthritis and other inflammation-related disorders based on its pharmacological effects in multiple tissues [9, 10]. Studies also have shown that SM could relieve postmenopausal symptoms and suppress bone resorption [11, 12]. Furthermore, serum containing SM induces MCF-7 cell proliferation and SM increases the estrogen-like effects of Qing'E formula [13, 14], which indicates that some components of SM involve in the activation of the estrogen receptor (ER). Tan IIA as a new member of the phytoestrogens, and its action might be activated by estrogen receptor (ER) in vascular endothelial cells [15]. Components of SM is clinically applied for treating postmenopausal symptoms, but concrete scientific data are lacking to evaluate whether SM is efficient in hormone replacement therapy, in contrast to the extensive studies on the estrogenic activity of isoflavones found in soybeans [16]. It is clear that the efficacy and molecular mechanisms of SM need to be elucidated for safer use of this promising therapy. Whether SM, as has been reported for phytoestrogens, causes few side effects or whether they are endocrine disruptors that endanger the uterus or vagina. In the present study, we describe the estrogenic effects of SM using *in vivo* models of immature and ovariectomized (OVX) mice along with *in vitro* studies to investigate its mechanism via estrogen receptor (ER) pathway. Besides, ER antagonist ICI182, 780, were studied to provide scientific data on SM and to identify potent agents for the prevention and treatment of postmenopausal syndrome.

## RESULTS

### Effect of SM on the estrus cycle

To characterize the estrogenic activity of SM on the reproductive tissues of immature mice and OVX mice, we compared the activity of SM with a synthetic estrogen, estradiol, and combine with the ER antagonist ICI182, 780 administration for elucidating the ER mechanism.

**Figure 1:**
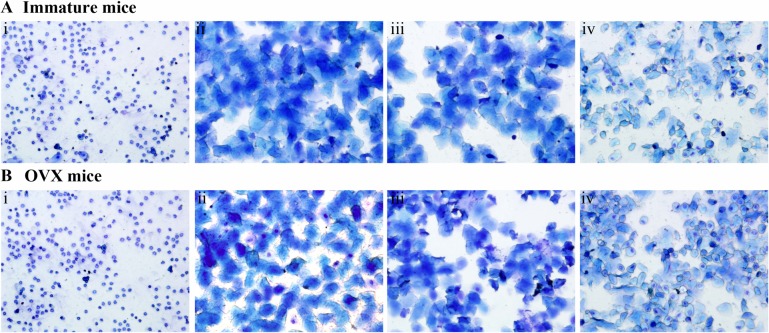
The effect of Salvia miltiorrhiza bunge (SM) on the estrous cycle ICI refers to the estrogen antagonist ICI182, 780 and E_2_ to 17β -estradiol. (**A**) The estrous cycle of the Immature mice, (i) the control group with untreated; (ii) Treated with estradiol (E_2_) ; (iii) Treated with Salvia miltiorrhiza bunge (SM) and (iV) Treated with Salvia miltiorrhiza bunge (SM) with estrogen receptor antagonist (ICI182, 780). (**B**) The estrous cycle of the OVX mice, (i) Ovariectomized (OVX) mice untreated; (ii) Sham group with untreated; (iii) Treated with Salvia miltiorrhiza bunge (SM) and (iV) Treated with Salvia miltiorrhiza bunge (SM) with estrogen receptor antagonist (ICI182, 780).

The estrus cycle of immature and OVX mice were daily monitored of vaginal epithelium cell smears. As shown in Figure 1A and 1B, untreated immature and OVX mice diestrus with presenting leukocytes in smears of vaginal epithelium. In contrast, the vaginal cells from the immature and OVX mice treated with SM at doses of 1.6, 3.2 g/kg or E_2_ became keratinized after 4 days and 10 days of treatment, respectively, which indicates advanced estrus in immature mice and restored estrus in OVX mice. Moreover, treatment with SM prolonged the estrous stage of the immature and OVX mice, suggesting very potent estrogenic activity. Whereas, in SM + ICI group, smears of the vaginal epithelium cells consisted of nucleated epithelial cells and less keratinocyte, indicating a proestrus, which had a similar effect to Co-treatment of SM + ICI group.

### Effect of SM on body, uterine and adrenal gland weights

**Figure 2:**
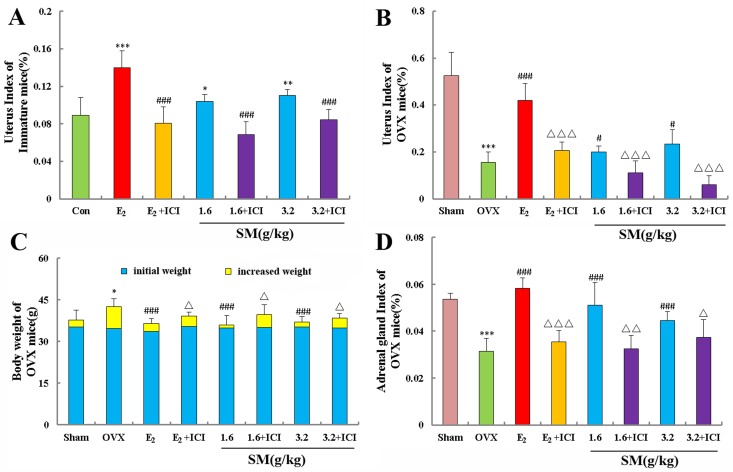
The effects of SM on uterine, body weights and adrenal gland (**A**) The uterine weights of immature mice were measured at the end of the 7-day treatment period. (**B**) The uterus index for ovariectomized (OVX) mice was measured at the end of the 4-week treatment period. (**C**) Body weights of OVX mice were measured once per week for 4 weeks. (**D**) The adrenal gland index of ovariectomized (OVX) mice was measured at the end of the 4-week treatment period. Data are the mean and standard deviation (SD) of samples from 10 mice. P values are for the one-way analysis of variance (ANOVA) comparing the treatment group with untreated mice. (**A**) ^**^*P < 0.001, ^*^*P < 0.01 and *P < 0.05 compared with the Con group; ^###^P < 0.001 compared with the SM group or E_2_ group; (**B**, **C**, **D**)^**^*P < 0.001 and *P < 0.05 compared with the Sham group;^###^P < 0.001 and ^#^P < 0.05 compared with the OVX group;Δ Δ Δ P < 0.001, Δ ΔP < 0.01, and ΔP < 0.05 compared with the SM group or E_2_ group.

Figure 2A, B showed that treatment with E_2_ resulted in significant estrogenic activity on the uterus. SM had modest stimulatory effects on the uterine weights of immature and OVX mice (all P < 0.05 or 0.01). A high dose of 3.2 g/kg of SM increased uterine weight by 1.2-fold and 1.5-fold compared to untreated immature and OVX mice, respectively. Co-treatment of SM or E_2_ + ICI induced a lower uterus index in immature and OVX mice than SM or E_2_ treatment alone (all P < 0.001).

The mice from all eight groups had similar initial mean body weights. At the end of the study, the mean body weight of mice in the OVX group was significantly higher than that of the sham group. A treatment with SM or E_2_ completely prevented the increase in body weight associated with E_2_ deficiency (Fig. 2C). The results suggested that SM could prevent body weight gain in postmenopausal women and had a better ability in reversing the body weight gain caused by ovariectomy than that of E_2_. As expected, the mean adrenal gland weight of OVX animals was significantly lower than that of sham controls as shown in Figure 2D. E_2_ treatment dramatically increased the adrenal gland weight of OVX mice compared with untreated control (p < 0.001). SM treatment had critical effects on adrenal gland weight gain, because a high dose of 3.2 g/kg of SM induced a 1.4-fold increase in adrenal gland weight compared with untreated OVX mice. ICI induced the decrease of adrenal gland index which was increased with E_2_ or SM treatment.

### Effect of SM on levels of serum E_2_, FSH and LH

**Figure 3:**
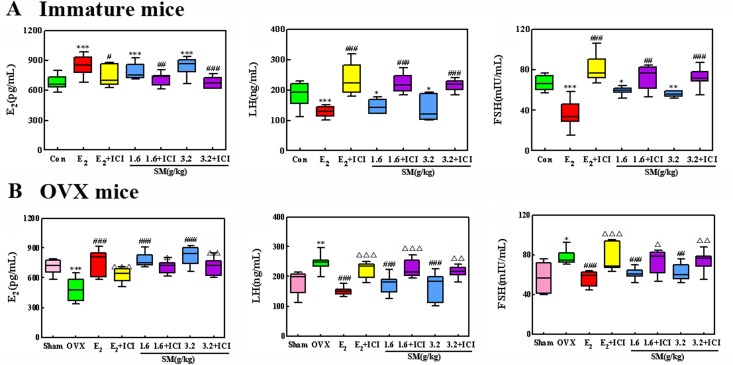
The effects of SM on serum estradiol (E_2_), luteinizing hormone (LH) and follicle-stimulating hormone (FSH) in immature and ovariectomized (OVX) mice (**A**) Serum levels of E_2_, LH and FSH from immature mice and (**B**) serum levels of E_2_, LH and FSH from ovariectomized (OVX) mice were measured at the end of the treatment period. Data are the mean and standard deviation (SD) of samples from 10 mice. P values are for the one-way analysis of variance (ANOVA) comparing treatment groups with untreated mice. (**A**) ^**^*P < 0.001, ^*^*P < 0.01 and *P < 0.05 compared with the Con group; ^###^P < 0.001;^##^P < 0.01 and ^#^P < 0.05 compared with the SM group or E_2_ group; (**B**)^**^*P < 0.001;^*^*P < 0.01 and *P < 0.05 compared with the Sham group;^###^P < 0.001 and ^##^P < 0.01 compared with the OVX group; Δ Δ ΔP < 0.001, Δ ΔP < 0.01, andΔ ΔP < 0.05 compared with the SM group or E_2_ group.

Immature and OVX mice have lower levels of serum E_2_ and higher levels of FSH and LH than mature and sham-operated mice, respectively. Treatment with SM or E_2_ significantly raised levels of serum E_2_ compared to those of untreated immature and OVX mice (all P < 0.001). The high dose of SM (3.2 g/kg) increased circulating E_2_ at 1.2- and1.9-fold compared with untreated immature and OVX mice, respectively. Meanwhile, SM treatment significantly decreased LH and FSH content in immature and OVX mice (P < 0.05, 0.01 or 0.001). More specifically, the high dose of SM (3.2 g/kg) resulted in 25%, 34% decrease in LH, and 16%, 23% decrease in FSH compared with untreated immature and OVX mice respectively. ICI significantly attenuated the increase of the serum E_2_ and decrease of LH and FSH in immature and OVX mice treated with SM or E_2_ (P < 0.05, 0.01 or 0.001). These results are illustrated in Figure 3.

### Effects of SM on the histology of uterus and vagina

**Figure 4:**
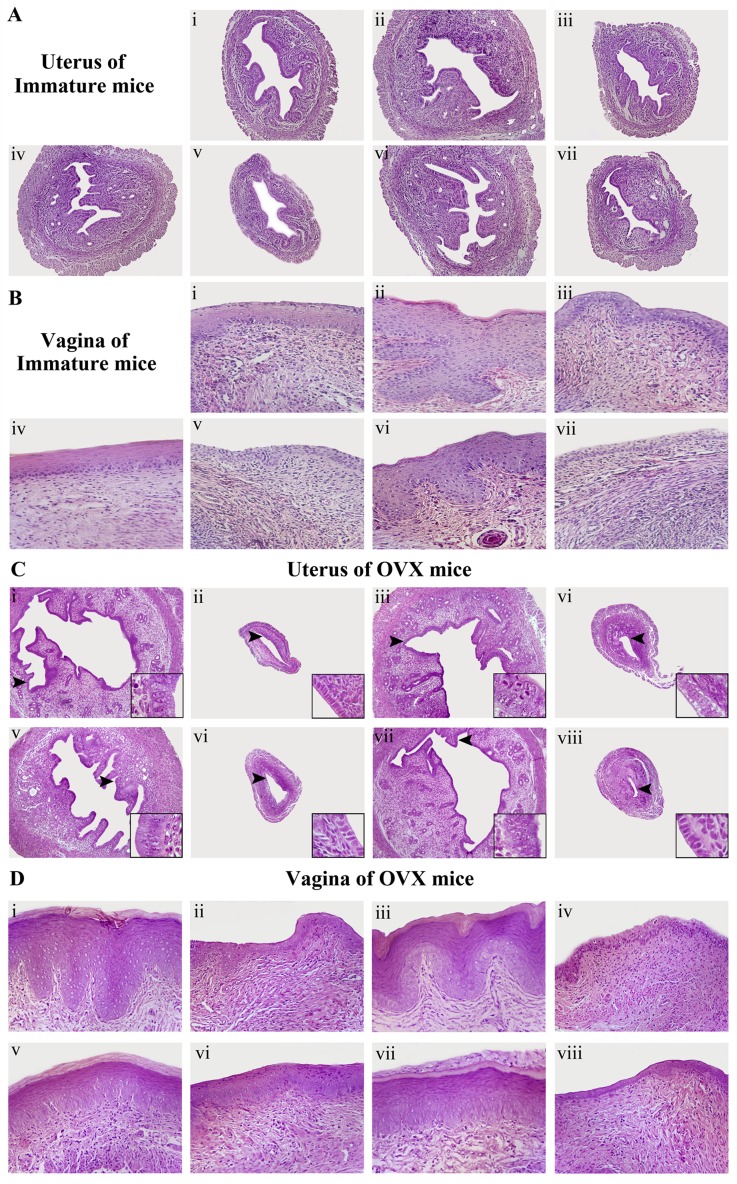
The effects of SM on the histology of the uterus and vagina in immature and ovariectomized (OVX) mice Representative photomicrographs taken at 200-X magnifiation of uterine in immature mice; 100-X magnifiation of uterine in ovariectomized (OVX) mice and 400-X magnifiation of vaginal sections. (**A**, **B**) are the histology of the uterus and vagina in immature mice. (**C**, **D**) are the histology of the uterus and vagina in ovariectomized (OVX) mice. The treatment groups in immature mice are shown: (i) control group; (ii) treated with E_2_; (iii) treated with E_2_ and ICI; (iv) treated with SM at 1.6 g/kg; (v) treated with SM at 1.6 g/kg and ICI, (vi) treated with SM at 3.2 g/kg; (vii) treated with SM at 3.2 g/kg and ICI. The treatment groups in OVX mice are shown: (i) sham-operated mice; (ii) untreated OVX mice; (iii) treated with E_2_; (iv) treated with E_2_ and ICI, (v) treated with SM at 1.6 g/kg; (vi) treated with SM at 1.6 g/kg and ICI; (vii) treated with SM at 3.2 g/kg; (viii) treated with SM at 3.2 g/kg and ICI.

As shown in Figure 4A, B, histological analysis of uterine sections revealed that treating with E_2_ or SM substantially induced the growth and development of the uterus and vagina in immature mice. These results in treated uteri samples were supported by observations of a thicker uterine endometrium, a higher number of glands and more extended glandular cavities than those of untreated samples. The endometrium was composed of single layered columnar epithelial cells, and no mitotic activity was detected in epithelial cells in untreated controls. In the SM-treated animals, endometrial cells were stimulated but no pathological signs were detected.

These morphologic findings of all animals were quantified and presented in Table 1. In the untreated vagina, 8∼11 cell layers were observed. Compared to untreated immature mice, the E_2_-treated animals (Fig. 4B ii) displayed a typical squamous multilayered epithelium, where approximately 10 ∼ 16 cell layers were observed in all 10 samples. In 1.6 g/kg SM-treated animals, epithelium thickness and the number of cell layers were augmented in some areas. The treatment with 3.2 g/kg SM (Fig. 4B vi) increased epithelial thickness and the number of cell layers (11∼13 layers). While, in SM + ICI group, the growth and development of uterus and vagina were decreased, with a similar effect to E_2_ + ICI group.

**Table 1:** Quantitative data of histological feature in uterus and vagina

Group	Uterus endometrial thickness (*μ*M)	Uterus endometrial glands numbers	Vaginal epithelium thickness (*μ*M)	Vaginal epithelium cell layers
Con	72.79±8.99	9.60±1.67	32.05±4.83	9.86±1.07
E_2_	95.50±20.36*	12.14±3.44	58.63±13.57**	13.14±1.86**
E_2_+ICI	68.41±8.33^##^	4.25±0.96^##^	10.34±2.95^###^	5.75±0.96^###^
SM1.6g/kg	84.63±6.82*	10.33±2.66	39.34±3.29*	10.20±1.92
SM1.6g/kg+ICI	56.86±12.75^###^	3.50±1.91^##^	12.65±5.80^###^	5.43±1.13^###^
SM3.2g/kg	88.98±16.37*	12.00±4.78	46.30±4.69**	11.75±0.96*
SM3.2g/kg+ICI	63.86±16.11^#^	7.00±3.07^#^	13.13±2.77^###^	5.38±2.46^###^

The data represent the mean ± standard deviation (*n =* 10), **p < 0.01 and *p < 0.05 compared with the Con group; ^###^*p* < 0.001, ^##^*p* < 0.001 and ^#^*p* < 0.001 compared with the SM group or E_2_ group.

As shown in Figure 4C, D, and Tables 2, 3, histological analysis of uterine sections revealed significant atrophy in the uterus of untreated OVX mice compared with sham controls, based on obvious degeneration of the cavities, endometrium and secretory glands. Treatment of OVX mice with E_2_ or SM at 1.6 or 3.2 g/kg substantially restored the atrophy of the uterus, with the thickening of the uterine endometrium, the increased number of glands and more extended glandular cavities compared with untreated OVX samples. The stromal cells of endometrial lamina propria were well organized and spindle shaped. Endometrial mitotic activity was group and no pathological found in 5 of 10 mice in the SM low dose treatment group, in 6 of 10 animals in SM high dose treatment signs were found. E_2_ (Fig. 4C iii) induced estrogenic features, causing the endometrial epithelium to become multilayered, hypertrophic and glands hyperplastic in 7 of 10 animals. The mitotic activity was present in endometrial cells in most animals at various degrees.

**Table 2:** Summary of the physiological and pathological findings in the uteri of OVX mice after treatment with E_2_ or SM

Group	Spindle-shape Lamina propria cells	Endometrial epithelium with mitosis	Hyperplastic/Hypertrophic glands
Sham	9/10	8/10	0/10
OVX	0/10	1/10	0/10
E_2_	8/10	9/10	7/10
E_2_+ICI	0/10	2/10	2/10
SM1.6g/kg	4/10	5/10	0/10
SM1.6g/kg+ICI	1/10	1/10	0/10
SM3.2g/kg	5/10	6/10	0/10
SM3.2g/kg+ICI	1/10	2/10	0/10

**Table 3:** Effects of treatment with E_2_ or SM on morphological features of vagina in OVX mice

Group	Cell layers			Keratinization	Vacuolization	
	1-6	>6 to ≤10	>10		Incipient	Clear
Sham	0/10	3/10	7/10	9/10	1/10	2/10
OVX	10/10	0/10	0/10	0/10	0/10	0/10
E_2_	0/10	4/10	6/10	10/10	6/10	1/10
E_2_+ICI	8/10	2/10	0/10	2/10	0/10	0/10
SM1.6g/kg	3/10	7/10	0/10	5/10	0/10	0/10
SM1.6g/kg+ICI	9/10	1/10	0/10	1/10	0/10	0/10
SM3.2g/kg	3/10	6/10	1/10	7/10	4/10	0/10
SM3.2g/kg+ICI	8/10	2/10	0/10	2/10	0/10	0/10

In the untreated vagina, compared with sham mice, the vaginal epithelium of OVX mice was atrophic, showing fewer cell layers and no cornification in 10 of 10 mice. The E_2_-treated animals displayed typical squamous multilayered epithelium, approximately 10∼15 cell layers were observed in all 10 samples and presented in Table 3. In 1.6 g/kg SM-treated animals, epithelium thickness and the number of cell layers were augmented in some areas, cornification was observed in 5 of 10 rats and no cytoplasmatic vacuolization was noted in all samples. Treatment with 3.2 g/kg SM (Fig. 4D vii) increased epithelial thickness and the number of cell layers. Cornification was found in 7 of 10 animals, and an incipient cytoplasmatic vacuolization of epithelial cells was observed in 4 of 10 rats. While, SM + ICI group demonstrated similar effect to the E_2_ + ICI group that reversing the atrophy of uterus and vagina were antagonized.

These studies suggest that SM has significant estrogenic potential in reproductive target tissues, which is weaker than that of the synthetic estrogen, estradiol. The estrogenic efficacy of SM was antagonized, when ICI was introduced to the formula. These data prompted further studies to elucidate the molecular basis of SM activities, implying that the effects of SM are stimulated through the activation of ERs.

### SM increased the expressions of ER subtype in uterus and vagina

**Figure 5:**
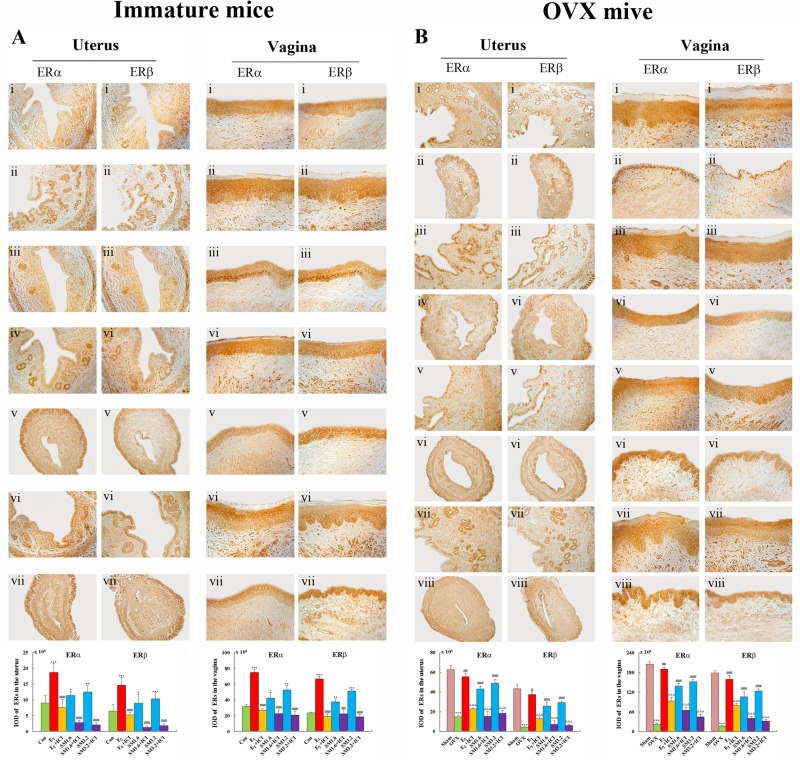
The effects of SM on the expressions of estrogen receptor ERα and β in the uterus and vagina ERs expressions were assessed by immunohistochemistry. Representative photomicrographs taken at 200-X magnifiation of uterine in immature mice; 100-X magnifiation of uterine in ovariectomized (OVX) mice and 400-X magnifiation of vaginal sections. (**A**) show expression of ERs in immature mice. Treatment groups are shown: (i) control group; (ii) treated with E_2_; (iii) treated with E_2_ and ICI; (iv) treated with SM at 1.6 g/kg; (v) treated with SM at 1.6 g/kg and ICI, (vi) treated with SM at 3.2 g/kg; (vii) treated with SM at 3.2 g/kg and ICI. (**B**) show the expression of ERs in the ovariectomized (OVX) mice. Treatment groups are shown: (i) sham-operated mice; (ii) untreated OVX mice; (iii) treated with E_2_; (iv) treated with E_2_ and ICI, (v) treated with SM at 1.6 g/kg; (vi) treated with SM at 1.6 g/kg and ICI; (vii) treated with SM at 3.2 g/kg; (viii) treated with SM at 3.2 g/kg and ICI. Data are the mean and standard deviation from 10 mice. P values are for the one-way analysis of variance comparing the treatment group with untreated mice. (**A**) ^**^*P < 0.001, ^*^*P < 0.01 and *P < 0.05 compared with the Con group; ^###^P < 0.001 and ^##^P < 0.01compared with the SM group or E_2_ group;^▴▴^P < 0.01 and ^▴^P < 0.05 compared with the ERα. (**B**)^**^*P < 0.001 compared with the Sham group;^###^P < 0.001 and ^##^P < 0.05 compared with the OVX group; Δ Δ ΔP < 0.001 compared with the SM group or E_2_ group.

Figure 5A, B shows representative sections of the expressions of ERα, ERβ in the uterus and vagina from each group and their corresponding quantitative analysis in immature and OVX mice. Treatment with either E_2_ or SM at any doses significantly increased ERα and ERβ expression in the uterus and vagina, compared with the untreated group in immature (P < 0.05, P < 0.01 or P < 0.001) or OVX mice (P < 0.01, P < 0.001), respectively. ERs in the uterus were expressed in the epithelial cells of the endometrium, interstitial cells, and smooth muscle cells. ERs in the vagina were expressed in the vaginal epithelium cells of squamous and smooth muscle cells. While, the expressions of ERα, ERβ in the uterus and vagina decreased in SM + ICI group, with a similar effect to E_2_ + ICI group. These data further support the hypothesis that SM mediates its activity *in vivo* through ERs.

### SM increased the protein and gene levels of ERs in uterus and vagina

**Figure 6:**
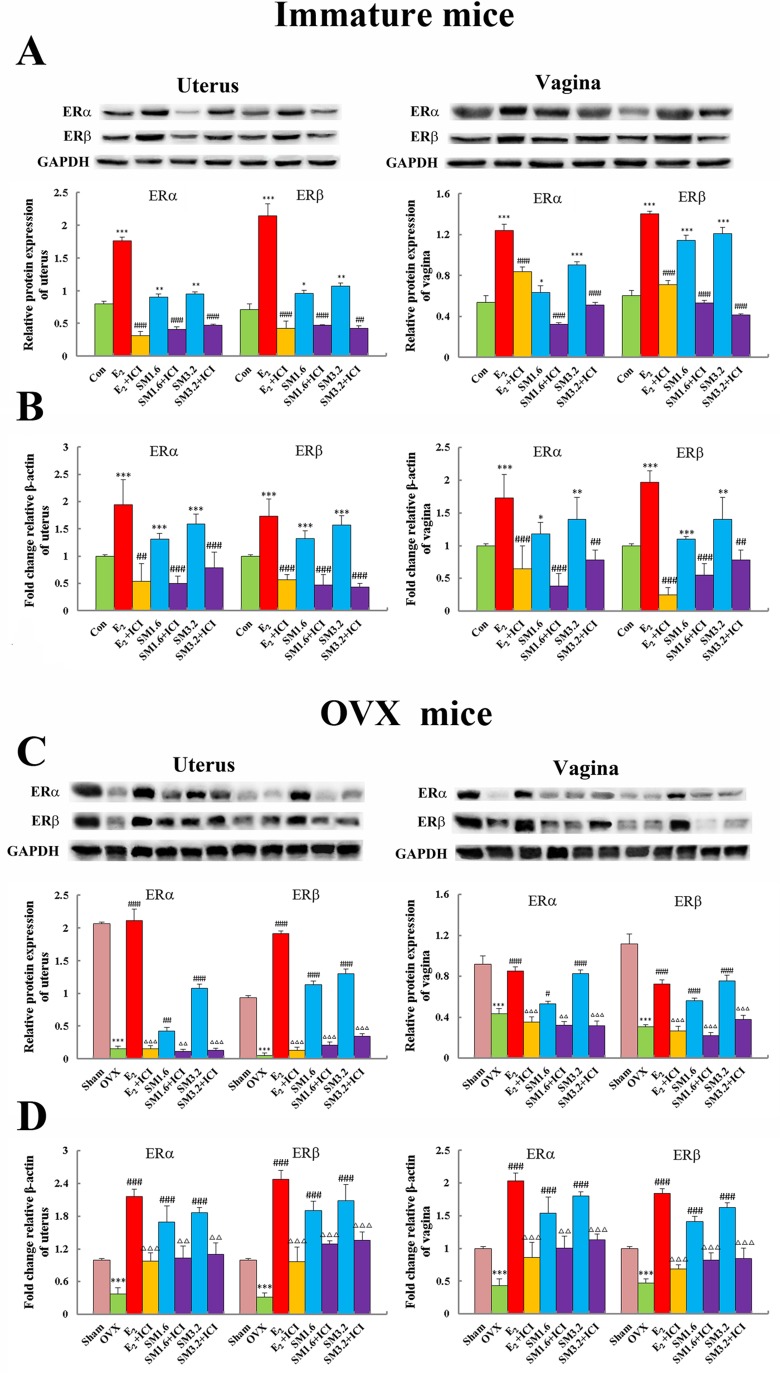
The effects of SM on the protein and gene expression of estrogen receptor ERα and ERβ in the uterus and vagina of mice Western-blot (**A, C**) and Realtime PCR (**B, D**) analysis was carried out as described in the Methods. Representative blots are shown above, and quantitative analyses are shown below. P values are for one-way analysis of variance (ANOVA) comparing treatment groups with untreated mice. (**A, B**) ^**^*P < 0.001, ^*^*P < 0.01 and *P < 0.05 compared with the Con group; ^###^P < 0.001 and ^##^P < 0.01compared with the SM group or E_2_ group;^▴▴^P < 0.01 compared with the ERα. (**C, D**)^**^*P < 0.001 compared with the Sham group;^###^P < 0.001, ^##^P < 0.01and ^#^P < 0.05 compared with the OVX group; Δ Δ ΔP < 0.001 and Δ ΔP < 0.01compared with the SM group or E_2_ group.

Western blot and real-time quantitative PCR were employed to examine ER subtype expressions on protein and mRNA levels in target tissues treated with SM. As shown in Figure 6A, a dose of 3.2 g/kg SM significantly increased the protein expression of ERα by 1.2-fold (p < 0.01) and ERβ by 1.5-fold (p < 0.01) compared to a 2.2- and 3.0-fold (both p < 0.001) increasing of ERα and ERβ induced by E_2_ versus untreated immature mice in the uterus, similar to the immunostaining results. The western blot results of immature mice vagina clearly showed that compared to the control group, treatment with SM (3.2 g/kg) stimulated levels of ERα and ERβ by 1.7- and 2.0-fold, respectively. Similarly, E_2_ induced a 2.30- and 2.31-fold increase in ERα and ERβ in the vagina (all p < 0.001). Meanwhile, the effects of SM and E_2_ on the gene expression of ERα and ERβ in target tissues were similar to those in protein levels as shown in Figure 6B. While, the protein and mRNA expressions of ERα and ERβ in the uterus and vagina decreased in SM + ICI group, with a similar effect to E_2_ + ICI group.

As shown in Figure 6C and D, compared with the sham group, both protein and gene expressions of ERα and ERβ were significantly decreased in the uterus and vagina of OVX mice (all p < 0.001). Treatment with either E_2_ or SM at the two different doses significantly increased protein and mRNA levels of ERα and ERβ in target tissues. More specifically, introducing 3.2 g/kg dose of SM to OVX mice increased protein expression of ERα by 5.99-fold (p < 0.001) and ERβ by 24.85-fold (p < 0.001) in the uterus, respectively, compared with those observed in untreated samples. This introduction also increased the protein expression of ERα by 3.2-fold (p < 0.001) and ERβ by 2.43-fold (p < 0.001) in the vagina, respectively, compared with untreated OVX mice. Meanwhile, compared with untreated OVX mice, the effects of SM and E_2_ on the gene expression of ERα and ERβ in target tissues were similar to those in protein levels as shown in Figure 6D. Besides, in SM + ICI group, the protein and mRNA expressions of ERα and ERβ in the uterus and vagina decreased, with a similar effect to E_2_ + ICI group.

### SM stimulated MCF-7 cell proliferation

**Figure 7:**
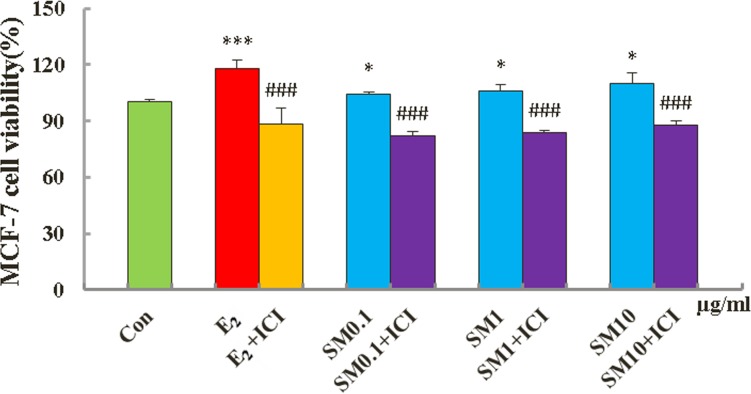
Effect of SM on viability of MCF-7 cells Cell proliferation was carried out as described in the Materials and Methods. Results are expressed relative to the growth of cells treated with 1% dimethylsulfoxide (DMSO). Data are the mean ± standard deviation of quadruplicate analyses, expressed relative to that of treatment with 0.1% DMSO. ***p < 0.001, *p < 0.05 compared to Con; ^###^p < 0.001, compared to SM or 0.01 μM E_2_.

To further investigate the molecular basis of SM activity, we used MCF-7 human breast cancer cells as the model because they are bound to estrogen for growth in monolayer culture. As shown in Figure 7, treatment with SM at dose levels of 0.1∼10 μg/mL and 0.01 μM 17 β-estradiol both stimulated proliferation of MCF-7, demonstrating estrogenic activity in the SM extracts. The SM + ICI mixture inhibited the proliferation of MCF-7 cells compared with SM treatment alone and resulted in significant differences (all P < 0.001), which is supported by an observation that the estrogenic activities of SM were significantly inhibited by the specific ER antagonist ICI182, 780.

### SM stimulated the binding effect of ERα and ERβ

**Figure 8:**
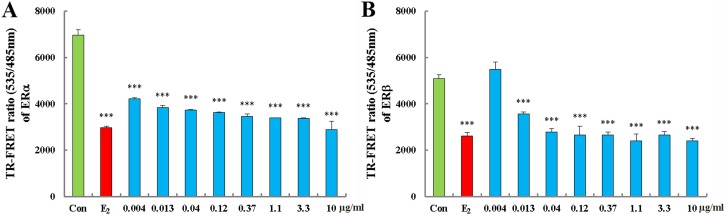
Effect of SM on ability of ERα binding (A) and ERβ (B) Each data point represents the mean ±standard of triplicate samples. ***p < 0.001 compared to Con.

The estrogenic activities of SM were significantly inhibited by the specific ER antagonist ICI182, 780 *in vivo* and *in vitro*. We next examined if SM could directly bind to ER using a TR-FRET ER competitive assay. As shown in Figure 8, SM could bind to human ERα and ERβ ligand binding domain (LBD) in the dose range of 0.013∼ 10 μg/mL. As the concentration increased, the combination was enhanced.

### SM induced both ERα and ERβ transcriptional activity

**Figure 9:**
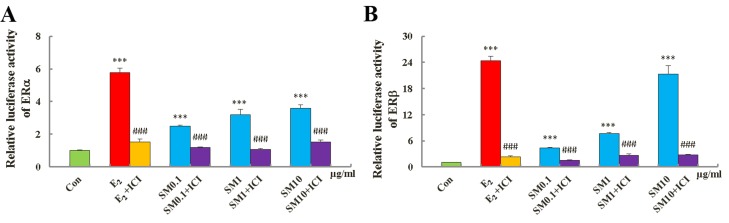
Activity of SM on estrogen receptor ERα (A) and ERβ (B) -estrogen response element (ERE) luciferases reporter gene expression Data are the mean ± standard deviation of quadruplicate analyses, expressed relative to that of treatment with 0.1% DMSO. P values are for one-way analysis of variance (ANOVA) comparing treatment groups with untreated mice. ^**^*P < 0.01, ^*^*P < 0.01 and *P < 0.05 compared with Con group; ^###^p < 0.001, compared to SM or 0.01 μM E_2_.

HEK 293 cells that had been stably transfected with the hERα/β -ERE-luciferase plasmid were used to measure the formation of functional hERα/β -ERE complexes in response to treatment with SM and individual compounds. Results are expressed relative to expression in DMSO-treated cells. 0.1∼10 μg/mL SM and 0.01 μM 17 β-estradiol both induced ERα and ERβ -ERE luciferase activities (Fig. 9). SM at 10 μg/mL induced a 2.58-fold increase in ERα and a 20.31-fold increase in ERβ luciferase activity. These effects were ablated when treatments were administered in the presence of the specific ER antagonist ICI 182, 780, resulting in 57% and 87% inhibition of ERα and ERβ-ERE-luciferase expression in cells treated with 10 μg /mL SM, respectively. These data indicate that SM clearly has estrogenic activity that is stimulated through the activation of ERs.

## DISCUSSION

It is well recognized that phytoestrogens have lower side effects compared with synthetic estrogen. Seeking effective phytoestrogens is an important and urgent issue in the prevention and treatment of postmenopausal syndrome [17]. In the present study, we investigated the estrogenic effects of SM using *in vivo* models of immature and ovariectomized (OVX) mice together with *in vitro* studies focusing on its mechanism via estrogen receptor (ER) pathway, and its combination with the ER antagonist ICI182, 780. The results showed that SM had a potent estrogenic activity, as indicated by promoting development of uterus and vagina in immature mice, restoring the estrus cycle and reversing the atrophy of reproductive tissues in OVX mice and no pathological signs were detected, increasing the expression of ER α and ERβ at proteinic and transcriptional levels in the reproductive tissues. Meanwhile, SM significantly increased serum estradiol, clearly decreased FSH and LH in circulation of immature and OVX mice. SM could bind to ERα and ERβ, and significantly induce ER-ERE luciferase expression. All activities were inhibited by the estrogen receptor antagonist ICI182, 780. This study demonstrates SM exerts estrogenic effects by stimulating biosynthesis of estrogen in circulation and increasing ERs expression in target tissues without side effects on reproductive tissues and through ER-ERE-dependent pathway.

Estrogens are mainly synthesized in the ovary. The increased serum estrogen concentration after the SM treatment suggests that the effect of SM might be activated through the hypothalamus-pituitary-ovary axis and stimulating the biosynthesis of estrogen in the ovary of immature mice. The adrenal gland becomes the principal tissue for secreting estrogen after ovariectomy [18]. The increased weight of adrenal gland and serum estrogen concentration with the SM treatment suggest that SM might take it action by activating the hypothalamus-pituitary-adrenal axis and stimulating the biosynthesis of estrogen in the adrenal gland. It is worth mentioning that the stimulation of estrogen synthesis in premature ovarian failure animals when they were administrated by other phytoestrogens [19]. Moreover, the increase in serum estradiol levels cause a decrease in FSH and LH production by inhibiting or the negative feedback of GnRH production in the hypothalamus [20, 21]. Our results suggested that SM induced higher estrogen release and inhibited the secretion of FSH and LH likely by negative feedback regulation.

Estradiol-17β (E_2_), a reproductive hormone, influences the growth, differentiation, and function of the female and male reproductive systems such as mammary gland, uterus, vagina, ovary, testes, epididymis, and prostate, and plays a vital role in body weight control [22]. In Post-menopausal women, estrogen deficiency is associated with increased probability of obesity [23]. Consistently, OVX rats that were deficient for estrogen and developed obesity could be reversed by E_2_ replacement therapy, which decreases food intake and increases energy expenditure [24]. Importantly, *in vivo* and *in vitro* experimental data have provided evidence that estrogen signaling decreases fat accumulation and body weight bound to ERα [25-26]. Our data showed that SM as a phytoestrogen also profoundly inhibited body weight increase and increased the expression of ERα in the target tissues of OVX mice.

In the uterus, E_2_ stimulates endometrial proliferation without the addition of progestin and this stimulation results in endometrial hyperplasia and could lead to neoplasia [27]. The vagina is another target for E_2_, since its epithelium is induced to undergo proliferation and cornification, which are the desired estrogenic effects because *Lactobacillus* use these cells to produce lactic acid to keep the vaginal milieu [28]. The uterus and vagina are known to be negatively influenced by estrogens used in HRT. Estrogens alone stimulate endometrial proliferation and may possibly lead to cancer [29-31], which has led researchers to a search for HRT alternatives, and plant-derived phytoestrogens have been vigorously promoted. Our data showed that endometrial epithelium become multilayered and hypertrophic and the glands hyperplastic in 7 of 10 OVX animals under 4-week E_2_ treatment (Fig. 4C iii). Whereas, endometrial cells of animals in the SM group were stimulated but no pathological signs were shown in immature and OVX mice, suggesting that SM might be safe for reproductive target tissues compared to E_2_. Under physiological conditions, the biological effect of estrogen is not only related to the level of estrogen, but also to the distribution and expression levels of the corresponding ERs in the target cells, ERα and ERβ [32, 33]. Estrogen and ERs are involved in the physiological function and stimulation of the female reproductive system. In our present study, SM significantly increased the expressions of ERα and ERβ in protein and gene levels in the target tissues, respectively. Estrogen mediates its actions by binding to the ER and inducing a major conformational change, which allow the estrogen-ER complex relocate to the nucleus to bind to its cognate DNA response element (ERE) located in the promoter/enhancer regions of target genes and regulation gene transcription [34]. In this study, we used a TR-FRET ER competitive assay illustrating that SM could bind to human ERα and ERβ ligand binding domain (LBD) in the dose range of 0.013∼10μg/mL. We also determined whether SM activated ER transcriptional activity (Fig. 8). In HEK293 cells co-transfected with ER and ERE-luciferase, SM induced a high level of ER transcriptional activity as measured by luciferase production (Fig. 9). The MCF-7 cell line expresses ERs and is dependent on estrogen for proliferation in monolayer culture [35]. We found that SM at the three different doses induced moderate proliferation of MCF-7 cells (Fig. 7). All agonist activities of SM *in vivo* and *in vitro* were strongly inhibited by the ER antagonist ICI182, 780, which suggests that SM exhibited estrogenic activities via the ERE pathway by interacting with the estrogen receptor.

In a recent report by Weng et al. [9], SM mediates through estrogen receptors to activate Akt and inhibit apoptosis effect of Leu27IGF-II-induced IGF-II receptor signaling activation in cardiomyoblasts. Fan et al. [36, 37] reported that tanshinone IIA is a new member of the phytoestrogens and its cardiovascular protection and anti-inflammatory activities were mediated by the ER activation. It is likely that the ability of SM to increase both ERs expression can be explained by the presence of multiple active components contained in the ingredient herbs that in combine exhibit polyvalent activities on ER activation in target tissues.

In a summary, all of these results strongly verified: SM has a potent estrogenic activity on reproductive target tissues without side effects. It exerts estrogenic effects by stimulating biosynthesis of estrogen in circulation and increasing ERs expression in target tissues and through ER-ERE-dependent pathway. These novel findings may shed light on the development of SM or its estrogenic compounds as an efficient and safe drug candidate in therapy of menopausal syndrome.

## MATERIALS AND METHODS

### In vivo studies

#### Animals and experimental design

The investigation has been conducted in accordance with the ethical standards and according to the Declaration of Helsinki and according to national and international guidelines and has been approved by Institute of Chinese Materia Medica, China Academy of Chinese Medical Sciences and all methods were carried out in accordance with the approved guidelines.

Immature mice model: Female, 21-day-old, immature mice (12 ± 2 g) and four-week-old, Kunming (KM) mice were purchased from Experimental Animal Center of Academy of Military Medical Sciences (Certifiate No. SCXK [Jun] 2012-0004). The immature mice were randomly assigned to seven groups: control, estradiol (E_2_, 0.1 g/kg), E_2_ plus estrogen receptor antagonist (ICI, 0. 005 g/kg), Salvia miltiorrhiza bunge (SM, 1. 6, 3. 2 g/kg), SM plus ICI, with 10 mice in each group.

Ovariectomized mice model: Four-week-old, Kunming (KM) mice maintained normal 5-day estrous cycles as confirmed by daily vaginal epithelium cell smear testing until the ovariectomy was performed. The dorsal ovariectomy was performed under general anesthesia using 0.3 mg/kg of chloral hydrate. All ovariectomized mice were checked by daily vaginal epithelium cell smear analysis, in which 5 consecutive days of leukocytes were indicative of constant diestrus and successful ovariectomy. In sham-operated negative controls, fat near the ovary was removed. The mice were randomly assigned to eight groups: sham operated (sham), ovariectomized without treatment (OVX), the rest of the six groups were the same with the immature mice. ICI group were given intraperitoneal injection, untreated control mice received, sham and OVX group distilled water only, the rest of the group were oral administrated once a day for consecutive 7 days and 4 weeks respectively. Dose calculations followed guidelines correlating the dose equivalents between humans and laboratory animals based on ratios of body surface area. All animals were maintained on a 12-hr light/dark cycle under constant temperature (24 *±* 2°C) and humidity (55 *±* 5%) and allowed free access to food and water.

#### Herbal preparation and analysis

Salvia miltiorrhiza bunge was purchased from Tongling Chinese Herbal Medicine Company, (Anhui, China) and identified and authenticated by an expert at the Institute of Basic Theory, China Academy of Chinese Medical Sciences. Preparation of water extract of SM: 32 g SM is cut into pieces, add 10 times distilled water, soak 30 min, the first time fried 60 min and filtered, the second time fried 40 min and filtered, then combine the filtrate and concentrate at 60°C rotary steam evaporator, and diluted with distilled water to the desired concentration of 80 g/L and 160 g/L. An high performance liquid chromatography (HPLC) method was developed for the representative chemical compositions of Rosmarinic acid (2.12125%), Salvianic acid A sodium (0.27125%), Salvianolic acid B (0.015%), Caffeic acid (0.01%), Lithospermic acid (0.0075%), Dihydrotanshinone I (0.0025%), Crytotanshinone (0.0025%), Tanshinone I (0.00625%), Tanshinone IIA (0.00125%).

#### Analysis of vaginal cornification, serum sex hormones and target tissues

Immature mice and OVX mice were monitored by daily vaginal epithelium cells smear testing during the 7-day and the last week of 4-week administration period, respectively. The vaginal lavage was fixed with 95% ethanol for 10 min and stained with methylene blue for 10 min [38]. Vaginal epidermal cells were observed by microscopy, and keratinized vaginal cells were taken as being indicative of estrus. All mice were sacrificed by decapitation. Blood was collected from the eyeball and 40 μL serum for analysis of estradiol (E_2_), follicle-stimulating hormone (FSH) and luteinizing hormone (LH) levels by enzymelinked immunosorbent assay (ELISA) (Beijing Xinfangcheng Biotechnology, China) [39]. The sensitivities ofthethree ELISA assays were 1.0 pg/ml, 1.0 mIU/ml and 1.0 ng/ml respectively and not soluble structural analogues with other cross-reaction, and all the intra-assay and inter-assay variation of each hormonal assay were less than 9% and 15%.

The uterus and vagina of immature mice and OVX mice and adrenal gland of OVX mice were removed and weighed. The left horns of the uterus and the upper portion of the vagina were stored at −80°C for analysis by western blot and Real-time quantitative Polymerase Chain Reaction, and the right horns of the uterus and the rest of vagina were fixed with 4% poly-oxymethylene for 24 h for staining with hematoxylin and eosin (H&E) and immunohistochemistry. The right samples were embedded in paraffin and prepared for cross-sections; sections 4 μm-thick were cut, mounted and stained with hematoxylin and eosin (H&E) for microscopy [40].

#### Immunohistochemistry

Tissue sections 4 μm-thick of uterus and vagina were mounted on polylysine-coated slides. The paraffin sections were dewaxed by a routine method and incubated for 10 min with 3% hydrogen peroxide (H_2_O_2_). Each section was incubated with blocking serum (Boster Biological Technology Co., Ltd) at room temperature for 30 min and then with primary rabbit anti-estrogen receptor-α antibody (dilution 1/20, Santa cruz Biotechnology) and a rabbit anti-estrogen receptor-β (dilution 1/30, Santa cruz Biotechnology), respectively, overnight at 4°C. Sections incubated in phosphate-buffered saline (PBS) without antibody served as negative controls. After incubation with bio-tinylated secondary antibody, sections were incubated with an avidin-biotin complex reagent containing horseradish peroxidase for 30 min. The sections were then stained with 3,3′-diaminobenzidine (DAB) (Boster Biological Technology Co., Ltd) [41]. The Image-Pro Plus 6.0 System image analysis system was used for quantitative analysis.

#### Western blotting

Uterus and vagina were resuspended in lysis buffer (50 mM Tris, pH 8.0, 150 mM NaCl, 5 mM EDTA, 0.1% sodium dodecyl sulfate (SDS), 0.5% NP-40) containing 10 mM phenylmethylsulfonyl fluoride (PMSF) and 2 mg/mL aprotinin. The protein was obtained to detect the levels of ERα and ERβ in target tissue by western blotting. The western blot protocol and semi-quantitative analysis were carried out as described [42]. The antibody of rabbit anti-ERα polyclonal antibody (dilution 1/200, Santa cruz Biotechnology) or mouse anti-ERβ monoclonal antibody (dilution 1/1,000, Abcam Biotechnology) was used. All experiments were done in triplicate. The relative quantity of each antibody was measured by Alpha Ease FC (Fluorchem FC2) software. The density ratio of protein to glyceraldehyde 3-phosphate dehydrogenase (GAPDH) (dilution 1/1,000, Cell Signaling Technology) was calculated from the band density.

#### Real-time quantitative Polymerase Chain Reaction (PCR)

After treatments, the total RNA of uterus and vagina was extracted with TRIzol reagent (Invitrogen, Carlsbad, CA, USA) according to the manufacturer's instructions. The total RNA (2 μg) was reverse transcribed to cDNA using the High Capacity cDNA Reverse Transcription Kit (Applied Biosystems Foster City, CA, USA), according to the instructions manual. The specific transcripts were quantified by quantitative real-time PCR using the Quanti Tect SYBR Green PCR Kit (QIAGEN K.K., Tokyo, Japan) and analyzed with an ABI 7500 real-time PCR system (Applied Biosystems, Foster City, CA, USA). Gene-specific primers were used for ERα (forward: CGCCTTCTAC AGGTCTAAT; reverse: GGTTCTTGTCAATGGTGC), ERβ (forward: CTGTGAGGTAGGAATGCGAAAC; reverse: GGTCTGGGTGATTGCGAAGA) and β-action (forward: CCTCTATGCCAACACAGTGC; reverse: CTGTAGAACGGTGTGGTCATC). The mRNA levels of ERα and ERβ were normalized to the β-action mRNA level. PCR was performed as 95°C for 10 min, followed by 40 cycles of 95°C for 30 s and 60°C for 1 min. The quantification data was analyzed with ABI Prism analysis software. The relative mRNA expression was calculated with the comparative *C*t method [43].

### In vitro studies

#### MTT assay of MCF-7 cell proliferation

The MCF-7 cell line was purchased from Xiehe Cell Research Institute of Peking Union Medical College (from the American Type Culture Collection [ATCC]), Three days before testing, the cells were cultured in DMEM without phenol red for purpose of depleting the intracellular estragon. The cells in logarithmic phase were digested by trypsin enzyme (0.05%), and cultured in DMEM without phenol red. Then, they were inoculated to the 96-well plate at 200 μL per well. After 24 hours, water extract of SM were respectively added into 0.001 mg/mL, 0.01 mg/mL, 0.1 mg/mL, 1 mg/mL. The 17 β-estradiol was also added into the plate at the dose of 10^−9^ M as well as the ICI182, 780 at the dose of 0.1 μM. 48 h after culturing, 5% MTT was add into every well at 10 μL. After 4h incubation, nutrient solution was absorbed and the DMSO was added per well at 150 μL. At 490 nm, MTT was used to measure the absorption value of the wells, and calculated the average absorbance value and proliferation rate [41].

#### ERs competitive ligand-binding assay

To confirm if SM could bind to ERS, LanthaScreen TR-FRET ERαand ERββcompetitive binding assay kits were used (Life Cat NO:A15883 and A15890). Briefly, series dilutions of SM (0.004, 0.013, 0.04, 0.12, 0.37, 1.1, 3.3 and 10 μ M) were competed with FluormoneTM GS1 Green for binding with terbium labeled ERs-LBD on a 384-well plate. One hour later after incubation at room temperature, the fluorescence inten-sity was detected on a microplate reader (Excitation: 340 nm; Fluorescein emission: 535 nm; Terbium emission: 485 nm; En-visionTM, PerkinElmer). The final data were shown by normalizing the signal of fluorescein to that of terbium.

#### Transfection and reporter assay of estrogen receptor-subtype selectivity

HEK 293 cells were stably transfected with human estrogen receptor α/β (hER α/β) and the estrogen response element (ERE) plasmid (kindly provided by Professor Yung-Chi Cheng, Yale University), and the luciferase reporter assay system from Promega (WI, USA) was used to evaluate the formation of functional ERα/β -ERE complexes. The cells were maintained and primed to minimize the effects of endogenous estrogens as described above and then seeded (1 × 10^5^ cells/100 μL/well) in 96-well plates. The test samples with or without ICI182, 780 and 17 β-estradiol were added to three replicate wells, as described for the MTT assay of MCF-7 cell proliferation, and was incubated for 24 h. Finally, the growth medium was carefully removed and 50 μL of lysis buffer per well was added, and the plate was rocked for 15 min. Twenty microliters of the detached cell solution was then transferred to a white micro well plate. Luciferase assay reagent (50 μL) was added to each well, and luciferase activity was measured immediately. Activity of the luciferase reporter gene was expressed relative to the DMSO control. Results reported are the mean ± standard deviation of three replicate determinations from a representative assay [44].

#### Statistics analysis

The software, SPSS version 11.0 for Windows (SPSS Inc., Chicago, IL, USA), was used for statistical analysis. All data was expressed as the mean standard deviation and were analyzed by one-way analysis of variance (ANOVA) followed by least significant difference (LSD) or the Dunnett's T3 test. Differences were considered statistically significant when the p-value was less than 0.05.
